# Statin use in setting of HIV infection

**DOI:** 10.1186/1471-2334-14-S2-S10

**Published:** 2014-05-23

**Authors:** Kenneth Lichtenstein

**Affiliations:** 1 National Jewish Health, Denver, Colorado, 80206, USA

## 

As HIV-infected individuals age due to improved antiretroviral therapy, they may be at increased risk for age-related co-morbidities such as cardiovascular disease (CVD). Increasing numbers of these individuals are initiating statins by meeting criteria for primary cardiovascular disease prevention [1].

Previous guidelines for the general population had recommended statin therapy based on 10-year cardiovascular risk (CV risk) with goal LDL-cholesterol (LDL-C) levels depending on the risk score. The latest guidelines have changed to identify four statin-requiring risk groups. They include: 1. Patients with known atherosclerotic cardiovascular disease. 2. Individuals with LDL-C ≥ 190 mg/dL (≥ 4.91 mmol/L). 3. Anyone age 40 to 75 with Type 1 or 2 diabetes mellitus (DM). 4. Individuals with a 10-year CV risk ≥ 7.5%. Statin therapy is then considered moderate intensity or high intensity when achieving a 30-50% reduction or > 50% reduction in LDL-C, respectively. The guidelines define the intensity of therapy that applies [2].

In HIV infection, incident cardiovascular events are higher than that of the general population [3-5]. Clinical judgment must be brought into play when deciding whether to follow the general population guidelines for calculation of 10-year CV risk and whether to select a lower risk value at which to start therapy. Also, there are three calculators: 1. Framingham risk calculation. 2. Pooled cohort risk calculation. 3. D*A*D risk calculation. To date, management guidelines in HIV-infection are lacking.

Providers must also be cognizant of the interactions of statins with protease-inhibitors and other drugs metabolized by the cytochrome CYP 3A enzyme and adjust the doses accordingly [6,7].

Finally, statins have recently been found to be associated with incident (DM). In the general population the benefits of statin therapy outweigh the risks of incident DM [8-13]. A study in an HIV-infected population demonstrated similar incidence of DM as compared to studies in the general population [14].

Statin therapy reduces CVD events in all at risk patients. Initiation of statin therapy in HIV-infection requires additional clinical judgment due to the increase risk of CVD events and drug interactions. The cardiovascular disease benefits of statins outweigh the risks of incident DM.
